# Population-Level Cell Trajectory Inference Based on Gaussian Distributions

**DOI:** 10.3390/biom14111396

**Published:** 2024-11-01

**Authors:** Xiang Chen, Yibing Ma, Yongle Shi, Yuhan Fu, Mengdi Nan, Qing Ren, Jie Gao

**Affiliations:** School of Science, Jiangnan University, Wuxi 214122, China; 6221204002@stu.jiangnan.edu.cn (X.C.); 6221204010@stu.jiangnan.edu.cn (Y.M.); 6221204012@jiangnan.edu.cn (Y.S.); 6231204003@stu.jiangnan.edu.cn (Y.F.); 6231204011@stu.jiangnan.edu.cn (M.N.); 6231204012@stu.jiangnan.edu.cn (Q.R.)

**Keywords:** trajectory inference, RNA velocity, Gaussian distribution, pseudo-time, single-cell data

## Abstract

In the past decade, inferring developmental trajectories from single-cell data has become a significant challenge in bioinformatics. RNA velocity, with its incorporation of directional dynamics, has significantly advanced the study of single-cell trajectories. However, as single-cell RNA sequencing technology evolves, it generates complex, high-dimensional data with high noise levels. Existing trajectory inference methods, which overlook cell distribution characteristics, may perform inadequately under such conditions. To address this, we introduce CPvGTI, a Gaussian distribution-based trajectory inference method. CPvGTI utilizes a Gaussian mixture model, optimized by the Expectation–Maximization algorithm, to construct new cell populations in the original data space. By integrating RNA velocity, CPvGTI employs Gaussian Process Regression to analyze the differentiation trajectories of these cell populations. To evaluate the performance of CPvGTI, we assess CPvGTI’s performance against several state-of-the-art methods using four structurally diverse simulated datasets and four real datasets. The simulation studies indicate that CPvGTI excels in pseudo-time prediction and structural reconstruction compared to existing methods. Furthermore, the discovery of new branch trajectories in human forebrain and mouse hematopoiesis datasets confirms CPvGTI’s superior performance.

## 1. Introduction

Trajectory Inference (TI) is a pivotal technique in single-cell analysis, reconstructing continuous biological processes from discrete experimental data. The goal of TI is to effectively rebuild the main trajectory that represents primary cellular states and their developmental branches, assigning a continuous value, known as pseudo-time, to each cell. Pseudo-time quantifies the position of cells within the trajectory, enhancing the identification of key cells involved in biological processes such as cell differentiation [1]. The advent of single-cell RNA sequencing (scRNA-seq) technology has significantly improved the resolution of cellular studies, enabling the investigation of developmental processes like embryogenesis [2] and cell differentiation [3] at the single-cell level [4]. Consequently, numerous TI methods have been developed to reconstruct potential cell trajectories [5,6,7,8,9], some of which focus solely on inferring cell pseudo-time [10].

However, scRNA-seq technology captures only static snapshots of cells, limiting the ability of traditional TI methods to identify trajectory directionality [11]. In recent years, RNA velocity modeling methods have emerged, allowing for the recovery of cell dynamic direction information and marking a new era in cell dynamics research [12]. Unlike the initial steady-state computational models, Bergen et al. developed a comprehensive mechanism to reproduce the RNA velocity of cells, resulting in a latent time with a more accurate differentiation direction [13]. Subsequently, several single-cell analysis methods incorporating RNA velocity have been presented, including trajectory inference, cell fate decision, and pseudo-time analysis [14,15,16]. Zhang et al. proposed CellPath, employing the Louvain community clustering algorithm in deep learning for pre-clustering, which employs the Louvain community clustering algorithm for pre-clustering and then defines meta cells to reconstruct the optimal path during cell development [14]. Lange et al. proposed CellRank, a method that combines cellular similarity with RNA velocity to reveal cell fate probabilities using Markov chain transition probabilities [15]. Mao et al. introduced LVPT, a pseudo-time inference model that incorporates RNA velocity and inertial probabilities to indicate the directional information of cells during a random walk [16]. RNA velocity has made predictive trajectory models possible by exploiting the balance between unspliced and spliced mRNA reads during the transcription process. However, existing TI methods based on RNA velocity often overlook issues of data sparsity and the distinction between true and false zeros [17], which can severely compromise the subsequent downstream analyses.

In this study, we introduce CPvGTI, a novel trajectory inference method based on Gaussian distribution, via predicting RNA velocities at the cell-population level. CPvGTI approximates the data as a Gaussian mixture distribution, which to some extent mitigates the negative impact of the sequencing data. Subsequently, the Expectation–Maximization (EM) algorithm is used to optimize the Gaussian Mixture Model (GMM) for data clustering. After refining the clusters to obtain cell populations, CPvGTI continues with the Gaussian assumption. It combines RNA velocity’s directed dynamic information to perform Gaussian Process Regression (GPR) on these populations. We demonstrate CPvGTI’s application and evaluate its performance on four real datasets and four simulated datasets with varying structures. The results validate CPvGTI’s ability to handle complex trajectory structures and outperform existing methods on these datasets.

## 2. Materials and Methods

### 2.1. Overview

**Figure 1:**
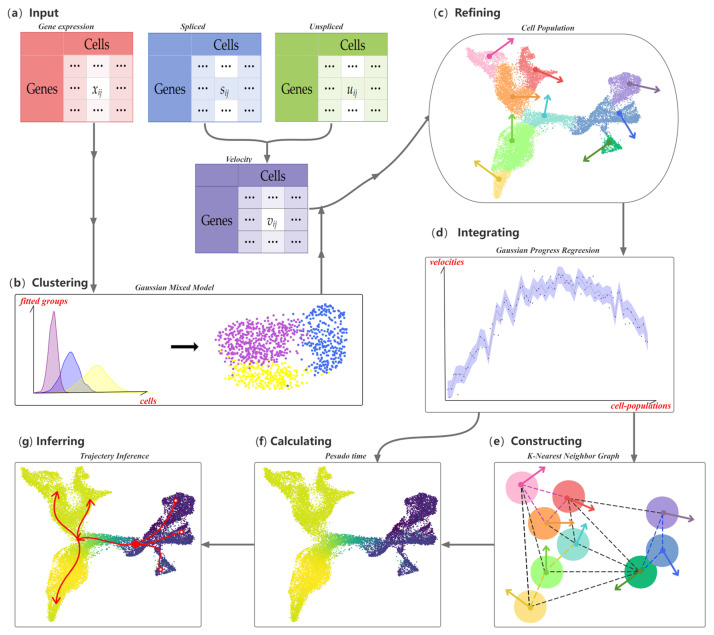
Overall workflow of the CPvGTI model: (**a**) The Input of CPvGTI, including a gene expression matrix, an unspliced mRNA count matrix, and a spliced mRNA count matrix; (**b**) Application of Gaussian Mixture Model clustering on gene expression matrix; (**c**) Refinement to segregate cells into Cell Population (CP), where RNA velocity is estimated first through the unspliced mRNA count matrix and spliced mRNA count matrix; (**d**) Gaussian Process Regression to integrate the gene expression and RNA velocity on CP level; (**e**) Construction of a K-Nearest Neighbor (KNN) graph with the directions provided by RNA velocity; (**f**) Calculation of pseudo-time for each cell. The darker the color, the earlier the inferred developmental time; (**g**) Trajectory inference, where the branches are marked with red arrows.

The workflow for CPvGTI is shown in Figure 1. The input data consist of three matrices: spliced count, unspliced count, and RNA velocity matrices. The spliced and unspliced count matrices are derived from the RNA velocities calculated using upstream velocity estimation methods such as velocyto [12] and scVelo [13]. We then apply a Gaussian distribution-based clustering model to identify cellular clusters. Within each cluster, we define a new hierarchical level—termed a cell population. Assuming a Gaussian distribution, we introduce the Gaussian Process Regression (GPR) model [18] to smooth the RNA velocities across each cell population. To establish the trajectory backbone, we construct a K-Nearest Neighbor (KNN) graph at the cell-population level. The Floyd–Warshall algorithm [19] is then utilized to explore potential developmental trajectories. Finally, the pseudo-time values of each cell are calculated by mapping each cell to its own population of cells.

### 2.2. Data Preprocessing and RNA Velocity Estimating

The mouse pancreatic endocrinogenesis dataset [3], mouse hematopoiesis dataset [20], and human cell cycle dataset [21] are accessible via the NCBI Gene Expression Omnibus (GEO) repository under accession numbers GSE132188, GSE140802, and GSE146773, respectively. The human forebrain dataset can be accessed from the Sequence Read Archive (SRA) database under NCBI, with the accession number SRP29388.

For a given dataset, CPvGTI uses three matrices as input: a gene expression matrix, an unspliced mRNA count matrix, and a spliced mRNA count matrix. Initial preprocessing of sequencing data is essential for subsequent analysis. This includes applying a minimum threshold for gene expression across cells, followed by centralization and normalization. Preprocessing steps can follow the scVelo protocol, utilizing functions such as *scv.pp.filter_and_normalize()* and *scv.pp.moments()*. RNA velocity, which connects measurements to the underlying dynamics of gene expression, provides directed dynamic information from single-cell transcriptomics. To facilitate the subsequent comparative analysis, the RNA velocity matrix used in this paper is calculated using scVelo.

### 2.3. Gaussian Mixture Model Clustering at the Cell Levels

The first input to the model is a gene expression count matrix derived from scRNA-seq, denoted as {*x_ij_*, i = 1,2,…,m, j = 1,2,…,n} with m cells and n genes. In the following text, the focus is primarily on the cellular level, hence the above matrix is simplified to **X** = {*x*_i_, i = 1,2,…,m}. Given the high heterogeneity and complexity of scRNA-seq data, we assume that it follows a specific statistical distribution model. The ZINB model, which has been widely studied in recent years, aligns to some extent with the characteristics of the data that exhibit a high number of zeros and sparsity [22]. However, the necessity of the zero-inflation condition, the overabundance of zero-expressed genes attributed to the negative binomial noise, and the high computational cost have been subjects of skepticism among researchers [23,24]. Recent studies suggest that it may be more appropriate to view cellular data as following a Gaussian distribution, as the Gaussian mixture framework can serve as a universal approximation for any continuous distribution [25]. Therefore, this paper posits that cells adhere to a Gaussian distribution, and this study utilizes a linear combination of multiple Gaussian distributions to fit the data distribution. GMM is employed to cluster cells in the following way:

(1) $$P_{data}(X)\approx P(X)=\sum_{k=1}^{K}\omega_{k}N(X\left|\mu_{k},\sigma_{k}^{2}\right.),$$

(2) $$N(X\left|\mu_{k},\sigma_{k}^{2}\right.)=\frac{1}{\sqrt{{\left(2\pi \right)}^{n}\cdot \left|\sigma_{k}^{2}\right|}}\exp\{-\frac{1}{2}{\left(X-\mu_{k}\right)}^{T}{\left(\sigma_{k}^{2}\right)}^{-1}(X-\mu_{k})\},$$

where $\sum_{k=1}^{K}\omega_{k}=1$. *ω_k_* represents the weight of the *k*-th Gaussian sub-model, and *μ_k_*, *σ*^2^*_k_* denote the mean and variance of the *k*-th sub-model, respectively. The parameters to be estimated can be written in the following way:

(3) θ=(ω,μ,σ).

To find the optimal values of these parameters, the EM algorithm is employed for iteration. The key to the EM algorithm lies in utilizing the posterior probability distribution of latent variables. Here, the latent variable is clearly the category *C_k_* to which the cells are assigned, and we define the latent variable **Z** in the following way:

(4) $$Z=\{z_{i},i=1,2,\ldots ,m\}\sim Multinimial(\Phi ),$$

where

(5) $$\Phi_{k}:P(z=C_{k}),\sum_{k=1}^{K}\Phi_{k}=1,$$

where *z* = *C_k_* indicates that the cell belongs to class *k*, and P(*z* = *C_k_*) is the probability distribution of the latent variable. After introducing the latent variable *z*, the probability distribution of each cell can be written as

(6) $$p(x)=p(x\left|\theta \right.)=\sum_{k=1}^{K}p(x,z=C_{k}\left|\theta \right.)=\sum_{k=1}^{K}p(z=C_{k}\left|\theta \right.)p(x\left|z=C_{k},\theta \right.),$$

where *p*(*z*=*C_k_*|**θ**) = *ω_k_*. Subsequently, the overall log-likelihood function is computed in the following way:

(7) $$l(\theta )=\log(\prod_{i=1}^{m}P(x_{i}\left|\theta \right.))=\sum_{i=1}^{m}\log(\sum_{k=1}^{K}P(z=C_{k}\left|\theta \right.)P(x_{i}\left|z=C_{k},\theta \right.)).$$

Now, the objective is to find the parameter that maximizes *l*(**θ**), that is,

(8) $$\hat{\theta }=\arg\max l(\theta ).$$

The essence of the EM algorithm lies in the continuous iteration of the E step and the M step, to obtain the optimal parameter $\hat{\theta }$. Here, the parameter **θ** at the *t*-th iteration is denoted as **θ^t^**. Thus, one iteration can be described as follows:

(9) $$\theta^{t+1}=\mathrm{argmax}E_{P(z\left|x,\theta^{t}\right.)}[\mathrm{logP}(x,z\left|\theta \right.)],$$

where *Q*(**θ**,**θ^t^**) is defined as log[P(*x*,*z*|**θ**)], that is *Q*(**θ**,**θ^t^**) = log[P(*x*,*z*|**θ**)]. Through the computation of the E-step, it can be obtained in this way:

(10) $$Q(\theta ,\theta^{t})=\sum_{i=1}^{m}\sum_{k=1}^{K}P(z_{i}=C_{k}\left|x_{i},\theta^{t}\right.)\mathrm{logP}(z_{i}=C_{k}\left|\theta \right.)\cdot P(x_{i}\left|z_{i}=C_{k},\theta \right.).$$

In the M-step, it is the aim to maximize the aforementioned expectation *Q*(**θ**,**θ^t^**). Through continuous iteration, the optimal parameters *ω*, *μ,* and *σ* are obtained in the following way:

(11) $$\omega_{r}^{t+1}=\frac{1}{m}\sum_{i=1}^{m}\gamma_{ir},$$

(12) $$\mu_{r}^{t+1}=\frac{\sum_{i=1}^{m}\gamma_{ir}x_{i}}{\sum_{i=1}^{m}\gamma_{ir}},$$

(13) $$\sigma_{r}^{t+1}=\frac{\sum_{i=1}^{m}{\left(x_{i}-\mu_{r}\right)}^{T}(x_{i}-\mu_{r})\gamma_{ir}}{\sum_{i=1}^{m}\gamma_{ir}},$$

where *γ_ir_* = P(*z_i_* = *C_r_*|*x_i_*,**θ^t^**). Based on the Law of Total Probability and Bayes’ Theorem, it can be reformulated in this way:

(14) $$\gamma_{ir}=\frac{P(x_{i}\left|z_{i}=C_{r},\theta^{t}\right.)\cdot P(z_{i}=C_{r}\left|\theta^{t}\right.)}{\sum_{k=1}^{K}P(x_{i}\left|z_{i}=C_{k},\theta^{t}\right.)\cdot P(z_{i}=C_{k}\left|\theta^{t}\right.)},r=1,2,\ldots ,k.$$

At this point, we have obtained the iterative parameters and posterior probabilities within GMM. Based on these, cells can be assigned to *k* clusters.

### 2.4. Gaussian Progression Regression Fitting at the Cell-Population Level

Obviously, a cell cluster is an ensemble formed by multiple cells connected in a certain way. The cells within this ensemble may exhibit similar behaviors and characteristics under specific conditions. However, such similarity does not preclude individual differences among cells. Even within the same cell cluster, there may be differences in directionality, function, or other aspects. Therefore, to mitigate the effects of neglecting the cell-specific information, the Cell Population (CP) is first defined before introducing RNA velocity. For each *k* in K, there exists a mapping χ:

(15) $$\chi :C_{k}\to \underset{s}{\cup }CP_{s},$$

where *s* represents the number of CP_s_ within each cluster.

Next, continuing with the assumption that the cell data follow Gaussian distribution, CPvGTI utilizes GPR to characterize the directional information of the CP. Gaussian Process is a type of stochastic process, which is a set of random variables that conform to a Gaussian distribution:

(16) $$f\sim \mathrm{GP}(\mu_{f},\sigma_{f}^{2}),$$

where *μ_f_* and *σ_f_*^2^ represent the mean and variance functions of the random variables, respectively. Initially, the observed dataset consists of the gene expression count matrix **X** and the velocity matrix **V**, which can be written as **D** = {(*x_i_,v_i_*), I = 1, 2,…, m}. The Gaussian regression model here can be formulated in the following way:

(17) V=f(X)+ε,

where *ε* represents Gaussian noise that follows a normal distribution N(0,*σ*^2^).

Based on the aforementioned definition of CP, a new count matrix can be obtained at the CP level, denoted as X*. The target of prediction is V*, which means that we need to add directional information to X*. More specifically, we need to use **D** and X* to predict V*, approximately, denoted as *f*(X*). According to the prior assumption, we can derive the joint Gaussian probability density function of *f*(**X**)and *f*(X*) in the following way:

(18) $$p(f(X),f(X*))=N\left(\begin{array}{cc} \left[\begin{array}{c} \mu_{f(X)}\\ \mu_{f(X*)} \end{array}\right], & \left[\begin{array}{cc} \Sigma & \Sigma_{*}^{Τ}\\ \Sigma_{*} & \Sigma_{**} \end{array}\right] \end{array}\right),$$

where **Σ** = **cov** (**X**, **X**). Similarly, it can be derived that

(19) $$\Sigma_{*}=\mathrm{cov}(X,X*),\Sigma_{**}=cov(X*,X*),\Sigma_{*}^{T}=cov(X*,X).$$

According to Bayes’ Theorem, *p*(*f*(**X**),*f*(X*)) = *p*(*f*(X*)|*f*(**X**))*p*(*f*(**X**)), our objective is to find *p*(*f*(X*)|*f*(X*)). Importantly, by utilizing the Schur complement to solve it, we can derive the following:

(20) $$p(f(X*)\left|f\right.(X))=N\left(\mu_{f}+\Sigma_{*}\Sigma^{-1}(f-\mu_{f}),\Sigma_{**}-\Sigma_{*}\Sigma^{-1}\Sigma_{*}^{T}\right).$$

Upon substituting the mean and covariance functions, this can be reformulated as follows:

(21) $$f(X*)=N(\mu_{f(X*)}+\Sigma_{*}\Sigma^{-1}\left[f(X)-\mu_{f(X)}\right],\Sigma_{**}-\Sigma_{*}\Sigma^{-1}\Sigma_{*}^{T}).$$

Finally, taking into account the noise distribution in the Gaussian process, it can be written as follows:

(22) $$V*=N(\mu *,\Sigma *),\mu *=\mu_{f(X*)}+\Sigma_{*}{\left(\Sigma +\sigma^{2}Ι\right)}^{-1}(V-\mu_{V}),\Sigma *=\Sigma_{**}+\sigma^{2}I-\Sigma_{*}{\left(\Sigma +\sigma^{2}I\right)}^{-1}\Sigma_{*}^{Τ}).$$

Generally, the covariance function, also referred to as the kernel function, plays a pivotal role. In this work, Gaussian kernel function, a type of radial basis function, is selected as the kernel function of the Gaussian process in the following way:

(23) $$\Sigma (x_{i},x_{j})=\sigma_{RBF}^{2}\exp(-\frac{{\left\|x_{i}-x_{i}\right\|}_{2}^{2}}{2l^{2}}).$$

Among these, *σ*_RBF_ and *l* are both hyperparameters. Together with the *σ* from the aforementioned Gaussian noise, they form a set of parameters to be estimated:

(24) $$\xi =(\sigma ,l,\sigma_{RBF}).$$

Similarly, maximum likelihood can be utilized to estimate the optimal **ξ**, consistent with the GMM approach. Ultimately, we obtain the direction of each CP level.

### 2.5. K-Nearest Neighbor Graph Construction at the Cell-Population Level

It is well known that trajectories are a research tool for learning cell phenotype features based on connected manifolds. Currently, there are many manifold learning methods for single-cell datasets, such as Multidimensional Scaling [26] and t-SNE [27]. To emphasize the overall topological structures and the relationships among cells, this paper prefers to use KNN graph as the graph construction method. This approach transforms cells from a data representation to a graph-based representation, emphasizing the topological connections. KNN graph is a graphical extension of the K-Nearest Neighbor algorithm. Existing methods characterize weighted undirected graphs based on the distances or the similarity information between cells. However, in this paper, considering the construction of RNA velocity and CP, the graph is a weighted directed graph that incorporates directional information. It is worth noting that the vertices of the graph are no longer cells but CPs, which enhances the detection of global topological structures.

First, we compute the adjacency matrix for the selected *k* neighbors (using the Neighborhood function). More importantly, we assign weights to each edge from CP*_s_* to CP*_q_*. These weights are primarily dictated by two differential components: expression difference *l_α_*(*s*,*q*) and distance difference *l_d_*(*s*,*q*). The expression difference *l_α_*(*s*,*q*) primarily reflects the variation on gene expression level and RNA velocity expression level between CP*_s_* and CP*_q_*, measured by cosine similarity, namely

(25) $$l_{\alpha }(s,q)=1-\cos\alpha ,$$

where

(26) $$\cos\alpha =\frac{{\left(CP_{s}-CP_{q}\right)}^{T}v_{s}}{{\left\|CP_{q}-CP_{s}\right\|}_{2}{\left\|v_{s}\right\|}_{2}}.$$

Obviously, the distance difference *l_d_*(*s*,*q*) primarily calculates the distance between CP*_s_* and CP*_q_*. Here, the Euclidean distance is used to represent it:

(27) $$l_{d}(s,q)=\frac{d_{s,q}}{d_{\max}}=\frac{{\left\|CP_{s}-CP_{q}\right\|}_{2}}{{\left(\sqrt{\left(CP_{s}-{CP_{q}}^{2}\right)}\right)}_{\max}}.$$

Similar to the work of Weng et al. [28] and Zhang et al. [14], we have ultimately determined that the weight of the edge is a nonlinear combination of the expression difference and the distance difference:

(28) $$e_{s,q}=\lambda {\left[l_{\alpha }(s,q)+\beta \cdot l_{d}(s,q)\right]}^{\lambda },$$

where *λ* and *β* are hyperparameters. Here, *λ* serves as a scaling factor, adjusting the balance between the disparities in expression and the differences in distance when calculating weights. Here, *β* acts as a distance factor, ensuring uniformity in the measurement of these two types of differences.

### 2.6. Possible Trajectories Detection and Pseudo-Time Analysis

After constructing the directed weighted graph, we proceed to identify potential trajectories within the graph. In this paper, the Floyd–Warshall shortest path algorithm is used to detect all possible trajectories. On one hand, compared to Dijkstra’s algorithm and the Bellman–Ford algorithm, the Floyd–Warshall algorithm can handle negative weight edges and solve all vertex pair problems at once. On the other hand, Dijkstra’s and Bellman–Ford algorithms are primarily used for single-source shortest paths, making them unsuitable for complex biological processes such as cell differentiation. However, the Floyd–Warshall algorithm can calculate the shortest paths between all vertex pairs. Essentially, the Floyd–Warshall algorithm is a process of continuous iterative looping over the graph nodes, which can be represented by the following iterative equation:

(29) $$\left\{\begin{array}{c} H_{s,q}^{\left(1\right)}=e_{s,q}\\ H_{s,q}^{\left(\nu +1\right)}=\min\left\{H_{s,q}^{\left(\nu \right)};H_{s,o}^{\left(\nu \right)}+H_{o,q}^{\left(\nu \right)}\right\} \end{array}\right..$$

In this equation, *o* represents the intermediate node CP*_o_* between CP*_s_* and CP*_q_*. More specifically, we can implement this through the following code (Algorithm 1):
**Algorithm 1** Floyd–Warshall Algorithm for shortest path detection**let** G = number of vertices in KNN graph **let** dist = G*G array of minimum distances initialized to ∞ **for** each vertex *g* dist[*g*][*g*] ← 0 **for** each edge (*s*,*q*) dist[*s*][*q*] ← *e*(*s*,*q*) **for**
*o* from 1 to G        **for** *s* from 1 to G              **for** *q* from 1 to G                      **if** dist[*s*][*q*] > dist[*s*][*o*] + dist[*o*][*q*]                           dist[*s*][*q*] ← dist[*s*][*o*] + dist[*o*][*q*]                       end if

Through the aforementioned shortest path detection algorithm, we can effectively calculate all possible paths between pairs of vertices.

Now that we have obtained trajectory paths covering each CP, the next step is to assign pseudo-time to the individual cells associated with each CP along the paths. Since the order of each CP is known, based on the RNA velocity information, we only need to sort the cells within each CP. Here, following the method of Zhang et al. [14], we similarly assume a smooth curve passing through the center of each CP. At each point on this curve Π(*t*), it holds that Π(*t_s_*) = CP*_s_*. A first-order Taylor expansion is used to approximate the pseudo-time *t*:

(30) $$\Pi \left(t\right)=\Pi \left(t_{s}\right)+\Pi^{\prime }(t_{s})(t-t_{s})+o(t-t_{s})=CP_{s}+v_{s}(t-t_{s})+o(t-t_{s}),$$

where o(*t* − *t_s_*) represents the higher-order term of Π(*t*). As *t* approaches t*_s_* infinitely, the above equation can be written as follows:

(31) $$\Pi \left(t\right)\approx CP_{s}+v_{s}(t-t_{s})=W\left(t\right),$$

where W(t) is clearly a linear function. Before calculating the pseudo-time for each cell, each cell is mapped to each CP:

(32) $${\hat{x}}_{si}=CP_{s}+\frac{(x_{si}-CP_{s})\cdot v_{s}}{{\left\|v_{s}\right\|}_{2}}\cdot \frac{v_{s}}{{\left\|v_{s}\right\|}_{2}}.$$

Using the linear function provided above, we calculate the pseudo-time t*_si_* for each cell *i*:

(33) $$\{t_{si}:{\hat{x}}_{si}=W(t_{si})\}.$$

After calculating the pseudo-time for cells in each CP, we normalize all the pseudo-times to ensure that the pseudo-time *t* of each cell falls within the range [0–1].

### 2.7. Evaluation Metrics on Simulated Datasets

To evaluate the performance of CPvGTI, we employ two metrics on the simulated datasets to assess the accuracy of trajectory reconstruction. Initially, we use the Kendall coefficient [29] to verify the correctness of the inferred cell order along each trajectory path. The Kendall coefficient is commonly used to measure the strength of the monotonic relationship between ordered variables, which is consistent with the mechanism of pseudo-time variables:

(34) $$\tau_{b}=\frac{\left|concord-discord\right|}{\sqrt{\begin{array}{cc} \left(concord+discord+concord_{t}\right) & \left(concord+discord+discord_{t}\right) \end{array}}}.$$

Here, *concord* and *discord* represent the number of concordant and discordant cell pairs, respectively, while *concord_t_* and *discord_t_* represent the number of ties in the two orderings. By setting an absolute value for the numerator, the value range of *τ_b_* is [0–1]. The closer the value of *τ_b_* is to 1, the more accurate the cell ordering.

Secondly, based on the evaluation framework proposed by Saelens et al. [5], we use the Spearman correlation coefficient to define the correlation between the estimated pseudo-time and the ground truth:

(35) $$\rho =\frac{\sum_{i=1}^{m}\left(true_{i}-\bar{true}\right)\left(\left(pred_{i}-\bar{pred}\right)\right)}{\sqrt{\sum_{i=1}^{m}{\left(true_{i}-\bar{true}\right)}^{2}\sqrt{\sum_{i=1}^{m}{\left(pred_{i}-\bar{pred}\right)}^{2}}}},$$

where *pred*(*i*) denotes the order of cell *i* in the pseudo-time ranking, and true(*i*) represents the order of cell *i* in the actual ranking, with *ρ* ranging from −1 to 1. The value closer to 1 indicates a higher consistency between the pseudo-time and the ground truth.

## 3. Results

### 3.1. Performance on Simulated Datasets with Different Structures

To quantitatively assess the performance of CPvGTI, we generate simulated data with four distinct topological structures using dyngen [30], including unspliced count, spliced count, and cell development time. These structures include a simple linear structure, a bifurcating structure with two distinct endpoints (a binary tree structure), a trifurcating structure with three different endpoints, and a composite structure featuring both bifurcations and cycles (a cycle tree). Through the reconstruction results of CPvGTI, it is easy to distinguish differentiation paths within these structures, including the successful separation of highly similar paths. Additionally, CPvGTI can not only reconstruct the topological structures, but also accurately infer the direction of each path, particularly in the composite structures.

**Figure 2:**
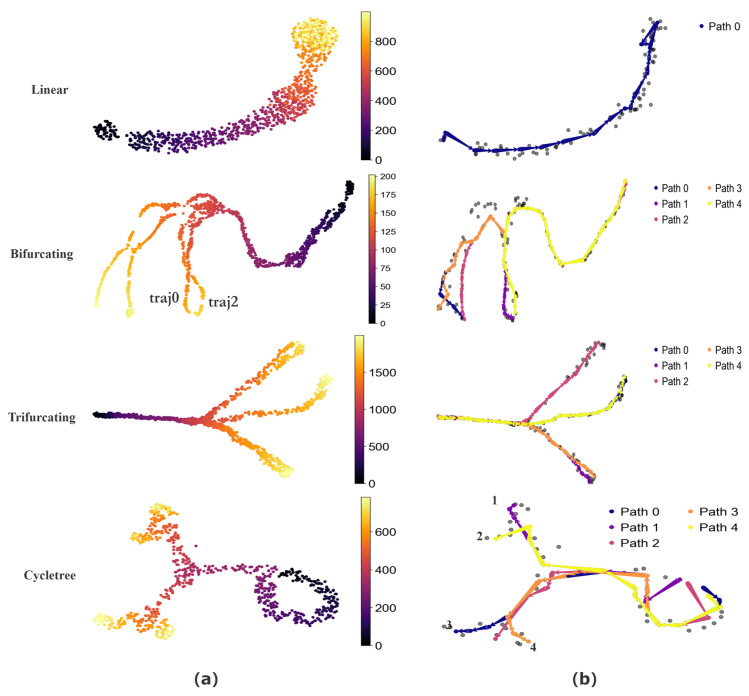
The performance of CPvGTI on simulated datasets: (**a**) The ground truth of four datasets with different structures. The color is labeled as the real developmental time simulated by dyngen; (**b**) The trajectories generated by CPvGTI. The corresponding branches are labeled by the legend.

The basic visualization of these structures can be seen in Figure 2a. The Bifurcating and Trifurcating sections exhibit some extremely similar differentiation paths in some branches, such as the ground truth traj0 and ground truth traj2 in the Bifurcating section of Figure 2a. Through the reconstruction results of CPvGTI (Figure 2b), it is clear that CPvGTI can successfully distinguish these very similar differentiation paths with the fewest paths. The Linear section and Cycletree section of Figure 2a simulate a small part of the cyclic structure, located in the upper right corner of the Linear section and the lower right corner of the Cycletree section, respectively. Clearly, CPvGTI can not only successfully reconstruct the topological structure of this part, but also correctly infer the direction of each path. Particularly, in the Cycletree section, it also represents another branch tree structure, including the four paths. Moreover, the starting point of all paths is on the cyclic structure, which is consistent with the ground truth.

To further compare the performance of CPvGTI with other methods, we employ dynmethods [5] to invoke different algorithms, including LVPT [16], CellPath [14], scVelo [13], and DPT [10]. Each method is tested on these four simulated datasets under the same experimental conditions. To clearly compare these methods with CPvGTI, we use the Kendall coefficient and the Spearman correlation coefficient as precision metrics to evaluate the accuracy of pseudo-time (see Table 1). As can be seen from Table 1, the Kendall and Spearman coefficients of CPvGTI have almost always achieved better results. Especially in complex structures, such as bifurcation and trifurcation, CPvGTI demonstrates good performance. This indicates that CPvGTI can infer pseudo-time that more closely matches actual cell development across different topological structures.

### 3.2. Reconstruction of Cell Cycle and Differentiation Trajectories in Pancreatic Endocrinogenesis

**Figure 3:**
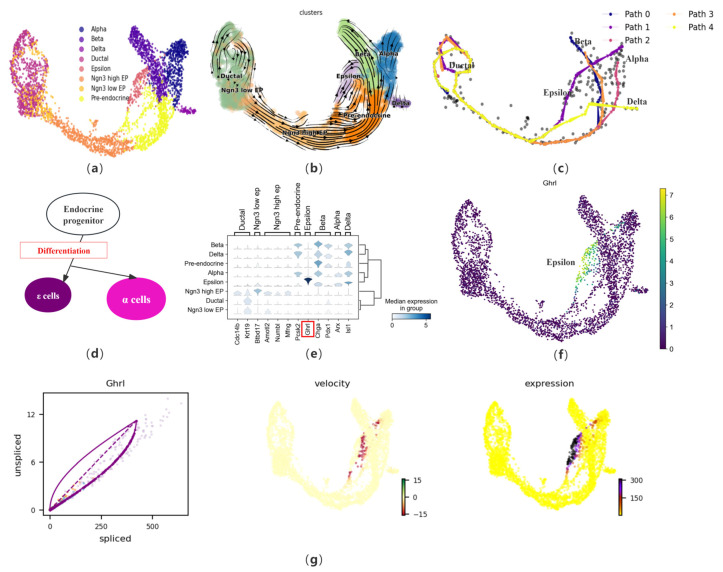
The performance of CPvGTI in mouse pancreatic endocrinogenesis: (**a**) The ground truth of the mouse pancreatic endocrinogenesis dataset visualized through scatter plot. Different colors represent different cell types; (**b**) The velocity manifold graph of the dataset calculated by scVelo; (**c**) The five trajectories generated by CPvGTI. Arrows are added to each lineage to indicate the direction of development; (**d**) The experimental results of Sakata et al. [31] on *epsilon* cells and *alpha* cells during the differentiation process of endocrine progenitors; (**e**) The stacked-violin plot of the expression on different terminal states (*alpha*, *beta*, *delta*, and *epsilon*) and their related marker genes. The position of the gene highlighted in red box can be visualized in (**f**); (**f**) The scatter plot of the marker gene *Ghrl* in *epsilon* cells; (**g**) The visualization of *Ghrl*, including the phase diagram, the RNA velocity plot, and the gene expression plot.

Considering the complex biological process of cell development, we first apply CPvGTI to a mouse pancreatic endocrine dataset containing four lineages. During the development of the pancreas, ductal cells, part of the pancreatic exocrine system, develop from the foregut endoderm to form endocrine progenitors (EP), which further proliferate and differentiate into islets of Langerhans. The EP begin to differentiate into two main cell types at specific stages of embryonic development: endocrine and exocrine cells. This dataset primarily focuses on the endocrine cell population of the islets, containing 3696 cells. The endocrine cells of the islets mainly include *alpha* cells, *beta* cells, and *delta* cells, which primarily produce glucagon, insulin, and somatostatin, respectively. Additionally, there are islet *epsilon* cells, identified as a novel endocrine cell population that produces Ghrelin. Ghrelin has been found to have a regulatory effect on other types of islet cells and may play a significant role in the development and differentiation of islets during the embryonic period. The cell type annotation results of Bastidas–Ponce et al. [3] is used as a reference for CPvGTI (Figure 3a). After preprocessing the dataset, CPvGTI reconstructs the biological processes (Figure 3c).

CPvGTI successfully captures the four lineages derived from the differentiation of progenitor cells. Notably, *epsilon* cells account for about 1% or less of the total number of mature islet cells, thus the developmental trajectory of this cell type has not been captured in many TI methods, such as CellPath and Vetra. However, CPvGTI successfully reconstructs the Path 1 with *epsilon* cells as the terminal state (Figure 3c). It can be found that Path 1 appears to partially overlap with the path of pancreatic *alpha* cells (Path 2). According to the research of Sakata et al. [31], there is indeed an overlapping part between *epsilon* cells and *alpha* cells during the differentiation process of EP (Figure 3d). In addition, by observing the velocity manifold graph of the dataset (Figure 3b), a potential cyclic structure can be identified at the ductal cells. CPvGTI captures the cyclic structure of the cell cycle (Figure 3c). It is known from the differentiation of islet cells that the starting point of each lineage should be the ductal cell. At the ductal cells in Figure 3c, there are multiple overlapping trajectories. Since the four types of secretory cells are all differentiated from the ductal cell, the starting point of all trajectories should be the ductal cells. The trajectory reconstructed by CPvGTI is consistent with the actual biological development process.

Subsequently, differential expression analysis of marker genes is conducted. First, we visualize the expression of different terminal states (*alpha*, *beta*, *delta*, and *epsilon*) and their related marker genes (Figure 3e). Specifically, regarding the marker gene *Ghrl* [32] in *epsilon* cells, we track and visualize its gene expression (Figure 3f) and RNA velocity expression (Figure 3g). It can be clearly observed that *Ghrl* shows an upregulated trend in the later stages of expression, including the corresponding RNA velocity map and gene expression map. This trend represents that during the differentiation process, *epsilon* cells regulate other endocrine cells (especially *beta* cells) by releasing Ghrelin. Ghrelin increases blood glucose levels by inhibiting insulin release from *beta* cells, participates in the growth and proliferation of *beta* cells, and prevents *beta* cell apoptosis, which has high research value for the treatment of diabetes. These findings further confirm the effectiveness of CPvGTI in trajectory reconstruction. We also compare CPvGTI with other methods. Vetra distinguishes the starting population of each terminal state at the ductal cell site but confuses the terminal state positions, including the failure to identify the *delta* cell trajectory (Figure S1a). On the one hand, at the starting point of the trajectory, that is, the ductal cell, CellPath does not capture the different trajectory starting points corresponding to different terminal states (Figure S1b). On the other hand, CellPath fails to capture the differentiation trajectory of *epsilon* cells, which is also an important deficiency. CytoPath successfully reconstructs the four differentiation trajectories corresponding to islet cells (Figure S1c). However, on the cyclic structure at the site of the ductal cell, it requires 8000 simulations to obtain a partial cyclic structure, compared to CPvGTI, which saves a lot of running costs. Similarly, LVPT fails to capture the cyclic structure due to the linear characteristics of the built-in algorithm (Figure S1d).

### 3.3. Multi-Directional Development Trajectory Reconstruction of Mature Neurons in Human Forebrain Dataset

**Figure 4:**
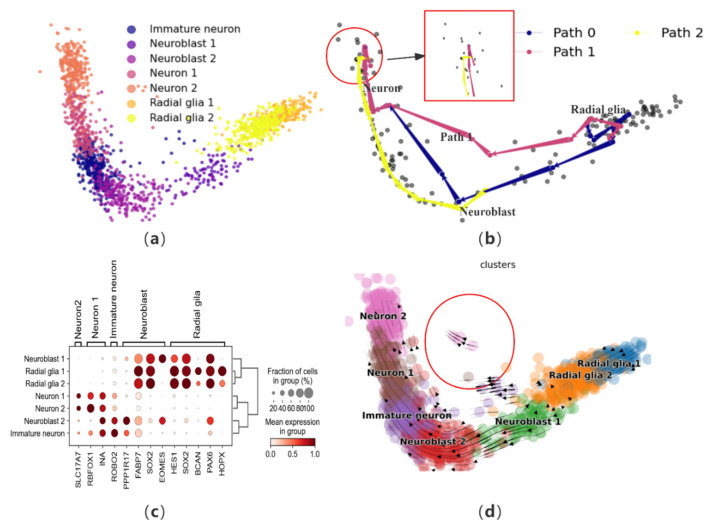
The performance of CPvGTI in the human forebrain dataset: (**a**) The ground truth of the human forebrain dataset visualized through scatter plot. Different colors represent different cell types; (**b**) The three trajectories generated by CPvGTI. The area within the red box seems to show that mature neurons show signs of reversion; (**c**) The dotplot of the expression of marker genes; (**d**) The velocity manifold graph of the dataset calculated by scVelo. The area within the circle seems to show that it exits another branch.

To further validate the effectiveness of CPvGTI, we perform trajectory reconstruction on a human forebrain glutamatergic neuron development dataset, comprising 1720 cells that outline the differentiation process of these neurons [12]. Initially, to gain an intuitive understanding of this neuronal differentiation process and terminal states, dimensionality reduction steps are conducted for visualization (Figure 4a). CPvGTI reconstructs the trajectory of glutamatergic neuron differentiation (Figure 4b), yielding a trajectory that adheres to an overarching linear structure. All paths align well with the progression of glutamatergic neuron differentiation, wherein radial glia cells transform into neuroblasts and subsequently mature into neurons.

In order to further assess the trajectories generated by CPvGTI, we employ marker genes for validation. Based on the human forebrain atlas created by Braun et al. [33], we visualize several key marker genes (Figure 4c and Figure S2a). The axes beneath the figure denote the genes, while those on the left and top represent the cells corresponding to each marker gene. By integrating the information from Figure 4c, the trajectories produced by CPvGTI are consistent with the known reality of neuronal development. By observing velocity manifold diagrams, there are cells that appear to have different differentiation trajectories (Figure 4d). Consistently, within the trajectory constructed by CPvGTI, there exists a branching Path 1 that aligns with this observation (Figure S2b). To investigate this, we explore the differentiation and development process of radial glial cells. These cells play a pivotal role in brain development, particularly in the genesis of neurons in the cerebral cortex. Typically, neuron generation mainly occurs through the differentiation of neuronal precursor cells via multiple intermediate steps. However, it has been demonstrated that radial glial cells also pass through neurogenic phases, functioning as neural progenitor cells [34]. Asymmetric division of radial glia cells gives rise to projection neurons, which ultimately differentiate into mature neurons in the inner layers of the cerebral cortex [35]. This implies that mature neurons can differentiate from either neuronal progenitor cells or radial glial cells. On the other hand, from Figure 4b (the area within the red box), it seems that mature neurons show signs of reversion. Hernández-Ortega et al. confirmed in their experiment that mature neurons do indeed re-enter the cell cycle [36]. In recent years, Poplawski et al. further confirmed that the developmental state of mature neurons can be reversed, marking a starting point for novel therapies against diseases like Alzheimer’s [37]. This groundbreaking discovery is consistent with part of the trajectory generated by CPvGTI. While CellPath is capable of replicating the original linear differentiation path, it fails to identify the trajectory related to neurogenesis (Figure S2c). LVPT encounters similar limitations, unable to capture the alternative differentiation path and the potential for mature neuron differentiation (Figure S2d). scVelo provides the latent time for differentiation, but there are discrepancies with the facts regarding the differentiation of radial glial cells (Figure S2e). The overall trend of pseudo-time inferred by Vetra is consistent with the trajectory inferred by CPvGTI (Figure S2f).

### 3.4. Reconstruction of Differentiation Trajectories of Multipotent Progenitor Cells in a Mouse Hematopoiesis Dataset

**Figure 5:**
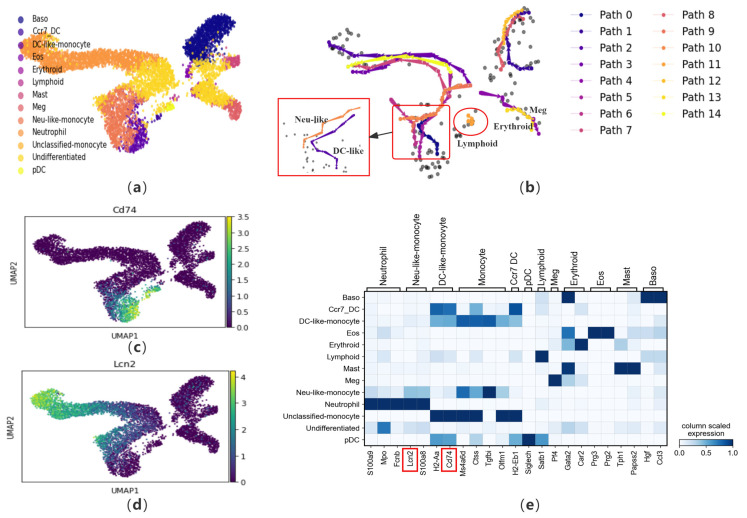
The performance of CPvGTI in mouse hematopoiesis dataset: (**a**) The ground truth of the mouse hematopoiesis dataset visualized through scatter plot. Different colors represent different cell types; (**b**) The fifteen trajectories generated by CPvGTI. Arrows are added to each lineage to indicate the direction of development. The figure marked by the large red box is a detailed representation of the figure indicated by the small red box. The red circle highlights the newly parts discovered through CPvGTI; (**c**) The matrixplot of the expression of marker genes; (**d**) The scatter plot of the marker gene *Cd74*. The lighter the color, the stronger the expression; (**e**) The scatter plot of the marker gene *Lcn2*. The lighter the color, the stronger the expression. The positions of the genes highlighted in red box can be visualized in (**c**,**d**).

To verify the performance of CPvGTI in handling multiple differentiation trajectories, we apply it to a mouse hematopoietic dataset [20]. During tissue renewal, stem cells and progenitor cells differentiate into mature cell types. This dataset contains 6555 cells that differentiate into 10 types of cells, including erythroid cells (Erythroid), megakaryocytes (Meg), basophils (Baso), mast cells (Mast), eosinophils (Eos), neutrophils (Neutrophil), monocytes (Monocyte), plasmacytoid dendritic cells (pDC), CCR7+ migratory dendritic cells (Ccr7DC), and lymphoid precursor cells (Lymphoid) (see Figure 5a). Among them, monocytes are further divided into undifferentiated monocytes, DC-like monocytes, and Neutrophil-like monocytes. CPvGTI successfully reconstructs the trajectories corresponding to each cell type (Figure 5b). For example, in Figure 5b, the area enclosed by the small box is the differentiation trajectory corresponding to various types of monocytes. We know that the differentiation paths of DC-like monocytes and Neutrophil-like monocytes are very similar. CPvGTI successfully distinguishes between these two differentiation paths (the large box on the left side of Figure 5b). On the other hand, in the lower right corner of the trajectory generated by CPvGTI, it represents the path of differentiation into erythroid cells and megakaryocytes. In fact, this part of the differentiation process is generally that multipotent stem cells first differentiate into megakaryocyte-erythroid progenitor cells (MEP), and then further differentiate into erythroid cells and megakaryocytes, respectively. CPvGTI successfully distinguishes these two parts of the cells and reconstructs the respective trajectories. In particular, the area enclosed by the red circle in Figure 5b is the differentiation trajectory of lymphoid precursor cells. Since lymphoid progenitors account for a relatively small proportion of the cells in this dataset, they are difficult to identify. To our delight, CPvGTI successfully reconstructs the differentiation path of lymphoid precursor cells. To elucidate the developmental processes along each trajectory, pseudo-time calculations are performed (Figure S3a).

Similarly, we visualize the marker genes (Figure 5e). To verify the accuracy of the Neu-like and DC-like trajectories reconstructed, we check them using the marker genes *Cd74* and *Lcn2* (Figure 5c,d). *Cd74* is a marker gene for DC-like monocytes. Upon comparison with Figure 5c, it becomes evident that *Cd74* is prominently expressed along the DC-like trajectory. Similarly, *Lcn2* is a marker gene for Neutrophil-like monocytes. In comparison to Figure 5e, *Lcn2* is also on the Neutrophil-like trajectory. In addition, we also check the marker genes in the trajectories corresponding to erythroid cells and megakaryocytes (Figure S3b,c). Comparing with the trajectory generated by CellPath, although it successfully distinguishes between the trajectories of DC-like and Neutrophil-like, it fails to capture the trajectory of lymphoid precursor cells and also fails to distinguish between erythroid cells and megakaryocytes (Figure S3d). By observing the pseudo-time generated by LVPT, it has been found that LVPT is unable to distinguish multiple lineages (Figure S3e). scVelo’s inferred latent time for cell development in this dataset shows discrepancies with the ground truth, especially in resolving the closely related trajectories of Erythroid and Megakaryocytes and Neu-like and DC-like monocytes (Figure S3f).

### 3.5. Reconstruction of the Cell Cycle from Single-Cell Proteomics

**Figure 6:**
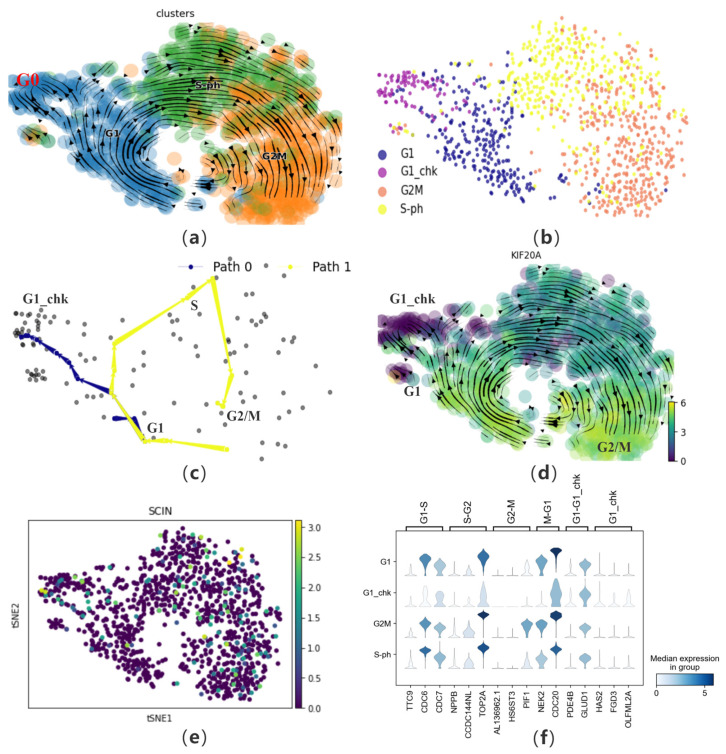
The performance of CPvGTI in human cell cycle dataset: (**a**) The velocity manifold graph of the dataset calculated by scVelo, where G0 and G1 is mixed; (**b**) The ground truth of the human cell cycle dataset visualized through scatter plot which is re-annotated. Different colors represent different cell types; (**c**) The two trajectories generated by CPvGTI. Arrows are added to each lineage to indicate the direction of development; (**d**) The velocity manifold graph of the cell cycle-dependent (CCD) protein KIF20A calculated by scVelo. The lighter the color, the stronger the expression; (**e**) The scatter plot of a protein unrelated to the cell cycle, SCIN. The lighter the color, the stronger the expression; (**f**) The stacked-violin plot of the expression on the marker genes.

To further investigate CPvGTI’s ability to reconstruct cell trajectories from multiple omics perspectives, we apply it to a human cell cycle dataset containing 1046 cells. Mahdessian et al. experimentally elucidated the differences between cells from a proteomic standpoint, by integrating single-cell transcriptomics and single-cell proteomics [21]. We follow the approach of Gupta et al. [38] to update the cell cycle annotations. The annotation basis is marker genes such as CCNA2, obtained by Xia et al. [39] through fluorescence labeling experiments, which are expressed at lower levels in the G0 phase (Figure 6a and Figure S4a). Therefore, the G0 phase is annotated as the G1_chk phase to more accurately validate the reconstructed trajectories (Figure 6b).

First, CPvGTI successfully reconstructs two trajectories, including a cycle and a branch (Figure 6c and Figure S4b). The cyclic structure reflects the cell cycle process, specifically the mitotic part. It can be observed that on Path 1, cells start from the G1 phase, go through intermediate phases, and eventually return to the G1 phase, forming a complete cycle. Path 0 represents another possible fate of cells in the cell cycle. Cells in Path 0 are in the G0 phase in the original dataset, which is a quiescent state. In this state, cells do not divide but will re-enter the cell cycle after receiving certain signals. Next, we attempt to analyze the performance of specific proteins in the mitotic cell trajectory from the perspective of single-cell proteomics (Path 1). Mahdessian et al. identified new proteins related to mitosis in their experiment. Taking KIF20A as an example, this is a newly discovered and experimentally proven mitosis-related protein, also known as a cell cycle-dependent (CCD) protein (Figure 6d). Clearly, we find that this protein is indeed present on Path 0, mainly in the G1 and G2M phases. Conversely, it is almost not expressed on Path 0. Additionally, we also verify a protein unrelated to the cell cycle, SCIN (Figure 6e). It is observed that SCIN is almost not expressed on Path 1 and also not present on Path 0. From the perspective of single-cell transcriptomics [40], it is necessary to visualize marker genes for each phase (Figure 6f). The left coordinate of the violin heatmap represents the four phases of the cell data, and the markers above correspond to the different transition phases of the marker genes. By observing the expression of these genes, genes with higher expression levels during cell division generally show a significant decrease in expression in the G1_chk phase, such as CDC6 and TOP2A.

The trajectories generated by CellPath can identify two branches but cannot correctly reconstruct the cell cycle structure (Figure S4c). LVPT cannot reconstruct cyclic trajectories and only calculates the corresponding pseudo-time (Figure S4d). Clearly, the calculation of pseudo-time by LVPT does not match the truth, mainly in identifying the starting point of the trajectory as G1_chk and failing to distinguish between the two branches. The reconstruction results of CytoPath largely agree with the trajectories reconstructed by CPvGTI (Figure S4e). Due to the inapplicability to cyclic trajectories, the pseudo-time inferred by Vetra significantly differs from truth (Figure S4f).

## 4. Conclusions and Discussion

Trajectory inference remains a formidable challenge in single-cell analysis, with the goal of elucidating the dynamic developmental processes of cells. High-throughput sequencing technologies have indeed made it possible to achieve high-resolution trajectories, but they can only capture static snapshot expression profiles, lacking directional information about dynamic development. The emergence of RNA velocity modeling methods has largely alleviated the limitations of trajectory reconstruction based solely on expression similarity. Nonetheless, we have observed discrepancies between RNA velocities and irregular sequencing data, which can impact the accuracy of trajectory inference. To counteract these issues, a new trajectory inference tool is introduced, CPvGTI, which incorporates RNA velocity directional information at the CP level, under the assumption of a Gaussian distribution.

CPvGTI processes the data manifold using a Gaussian mixture to fit the data manifold, ensuring data order while retaining the characteristics of the data. To capture the specific information of RNA velocity that may exist, it employs Gaussian process regression to infer directionality at the CP level, instead of the cluster level. When tested on simulated datasets, CPvGTI outperforms the compared methods (Table 1), including DPT, scVelo, LVPT, and CellPath. Especially on complex datasets, CPvGTI can capture interwoven branches (Figure 2). In real datasets, CPvGTI also reconstructs some local paths that are not easily detected. For instance, in the mouse pancreatic endocrine dataset, CPvGTI reconstructs four lineages corresponding to four types of secretory cells, including pancreatic *epsilon* cells (Figure 3). Methods such as Vetra and LVPT did not identify the developmental branch of *epsilon* cells (Figure S1). Similarly, in the mouse hematopoiesis dataset, due to the very small proportion of lymphoid progenitor cells in the entire dataset, most TI methods classify the lymphoid progenitor cells into other cell populations or fail to recognize them (Figure S3). CPvGTI not only successfully identifies the lymphoid progenitor cell population but also reconstructs a clear trajectory for them (Figure 5).

In summary, CPvGTI has demonstrated its exceptional capability in trajectory reconstruction, underpinned by the overarching assumption of a Gaussian distribution. Indeed, we believe that the Gaussian framework and the concept of Cell Population inherent to CPvGTI will offer novel perspectives in single-cell data analysis. With the continuous advancements in single-cell sequencing technologies [41], we anticipate that CPvGTI will reveal its potential in a broader range of applications, including but not limited to the inference of cellular developmental trajectories, the analysis of cellular state transitions under disease conditions, and the study of cellular heterogeneity [42]. As single-cell multi-omics data integration technology continues to advance [43,44], we are confident that CPvGTI will play an increasingly vital role in future biomedical research.

## Figures and Tables

**Table 1:** Evaluation of TI methods across different cellular structures using Kendall and Spearman rank correlation coefficients. The bolded values represent the highest correlation coefficient scores for each structure.

Metrics	Structures	CPvGTI	LVPT	CellPath	scVelo	DPT
Kendall	Linear	**0.928784**	0.659118	0.854334	0.914235	0.556036
	Bifurcating	**0.944409**	0.262516	0.809100	0.789165	0.150742
	Trifurcating	**0.937211**	0.452521	0.854667	0.847968	0.630946
	Cycletree	**0.988250**	0.548864	0.936341	0.729273	0.686827
Spearman	Linear	**0.988360**	0.629686	0.899774	0.984890	0.589317
	Bifurcating	**0.994725**	0.319819	0.933391	0.893939	0.367037
	Trifurcating	**0.993466**	0.554871	0.959936	0.955595	0.800530
	Cycletree	**0.999361**	0.780334	0.927419	0.894422	0.880985

## Data Availability

CPvGTI is written in Python and available at https://github.com/XxC2X7/CPvGTI (accessed on 30 October 2024).

## Supplementary Materials

The following supporting information can be downloaded at: https://www.mdpi.com/article/10.3390/biom14111396/s1, Figure S1: Results of baseline methods on the pancreatic endocrinogenesis dataset, related to Figure 3; Figure S2: Results of baseline methods on the human forebrain dataset, related to Figure 4; Figure S3: Results of baseline methods on the mouse hematopoiesis dataset, related to Figure 5; Figure S4: Results of baseline methods on the human cell cycle dataset, related to Figure 6.
