# Supplementary material for: Population-Level Cell Trajectory Inference Based on Gaussian Distributions
Source: Biomolecules. 2024 Nov 1;14(11):1396. doi: 10.3390/biom14111396 (PMC11592043; doi:10.3390/biom14111396)
Supplement: Supplementary file 1 [file biomolecules-14-01396-s001.zip › Supplementary Data.pdf]

Population-Level Cell Trajectory Inference based on Gaussian Distributions

Xiang Chen [1], Yibing Ma [1], Yongle Shi [1], Yuhan Fu [1], Mengdi Nan [1], Qing Ren [1], Jie Gao [1*]

[1]School of Science, Jiangnan University, Wuxi 214122, China
*Correspondence: gaojie@jiangnan.edu.cn ; Tel.: +86-510-85912033

Supporting information to "Population-Level Cell Trajectory Inference based on Gaussian Distributions"

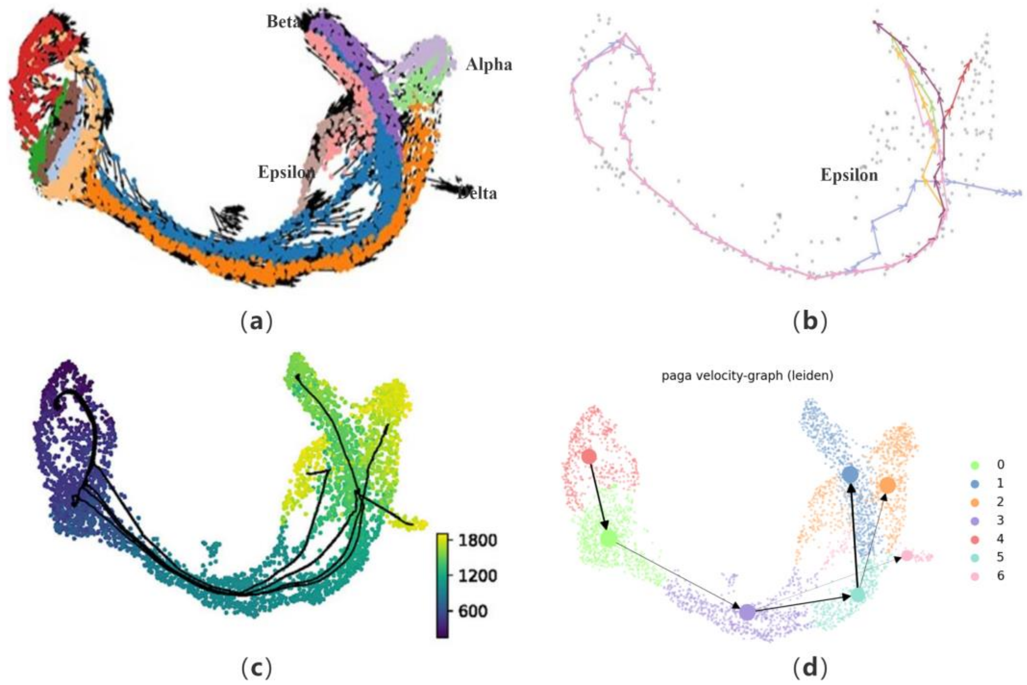

Figure S1. Results of baseline methods on the pancreatic endocrinogenesis dataset, related to Figure 3: (**a**) The result of Vetra on the pancreatic endocrinogenesis dataset; (**b**) The result of CellPath on the pancreatic endocrinogenesis dataset failing to identifying the *epsilon*-related lineage; (**c**) The result of CytoPath on the pancreatic endocrinogenesis dataset; (**d**) The result of LVPT on the pancreatic endocrinogenesis dataset.

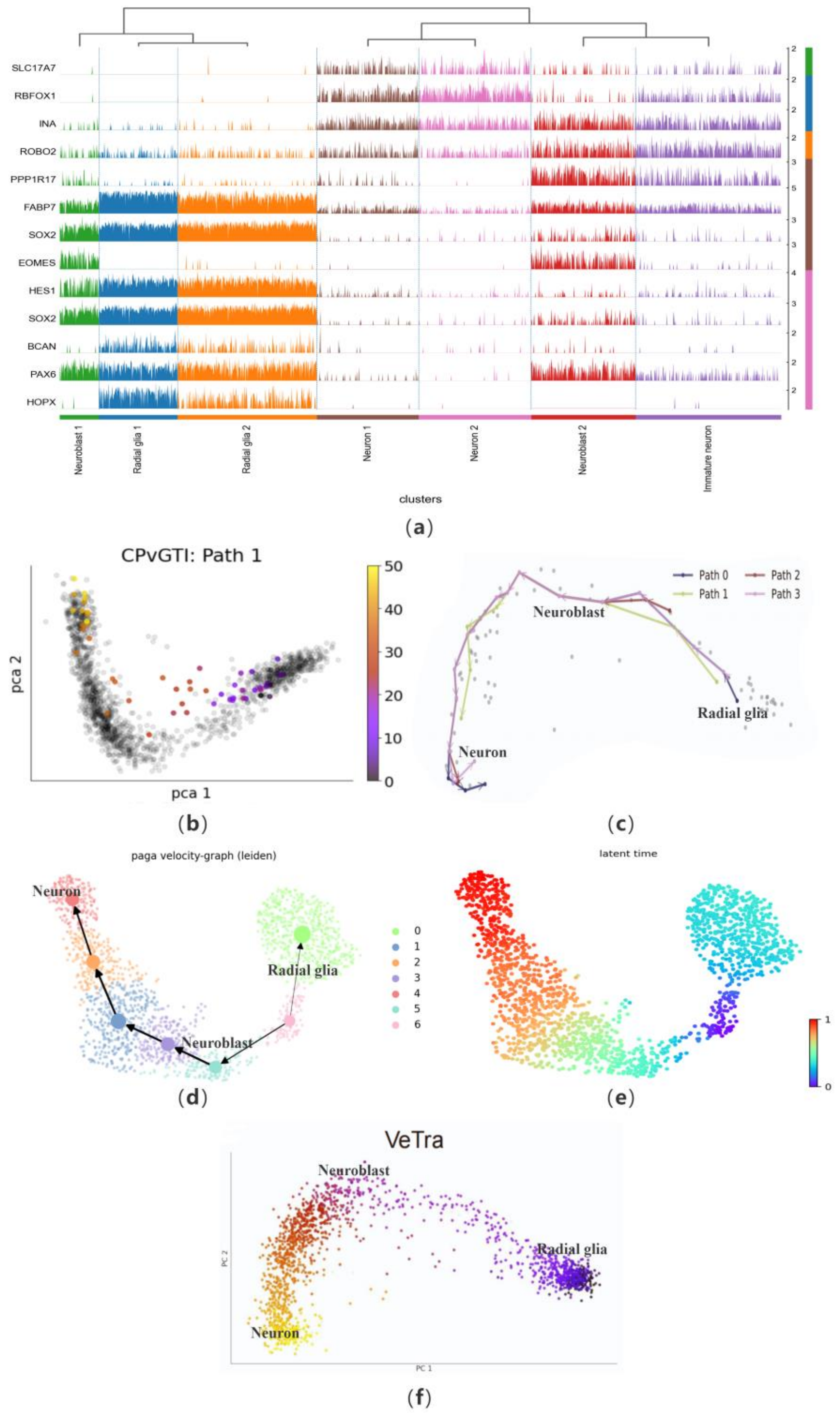

Figure S2. Results of baseline methods on the human forebrain dataset, related to Figure 4: (**a**) The tracksplot of several key marker genes; (**b**) The trajectory generated by CPvGTI on the human forebrain dataset, where the branching Path 1 is another direction of the differentiation; (**c**) The result of CellPath on the human forebrain dataset; (**d**) The result of LVPT on the human forebrain dataset; (**e**) Pseudotime result of LVPT on the human forebrain dataset; (**f**) The result of Vetra on the human forebrain dataset.

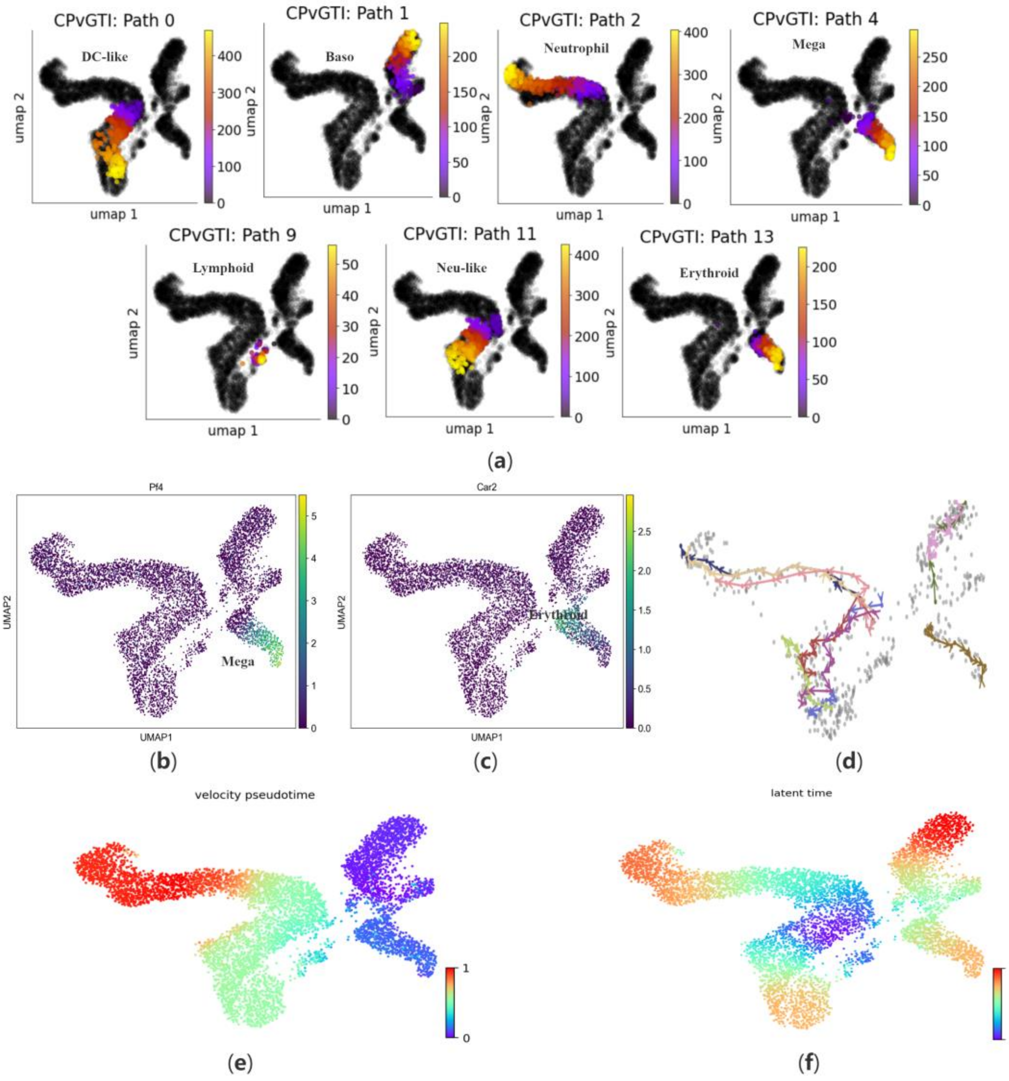

Figure S3. Results of baseline methods on the mouse hematopoiesis dataset, related to Figure 5: (**a**) The pseudo-time inferred by CPvGTI; (**b**) The scatter plot of the marker gene Pf4. The lighter the color, the stronger the expression; (**c**) The scatter plot of the marker gene Car2. The lighter the color, the stronger the expression; (**d**) The result of CellPath on the mouse hematopoiesis dataset; (**e**) Pseudo-time result of LVPT on the mouse hematopoiesis dataset; (**f**) Pseudo-time result of scVelo on the mouse hematopoiesis dataset.

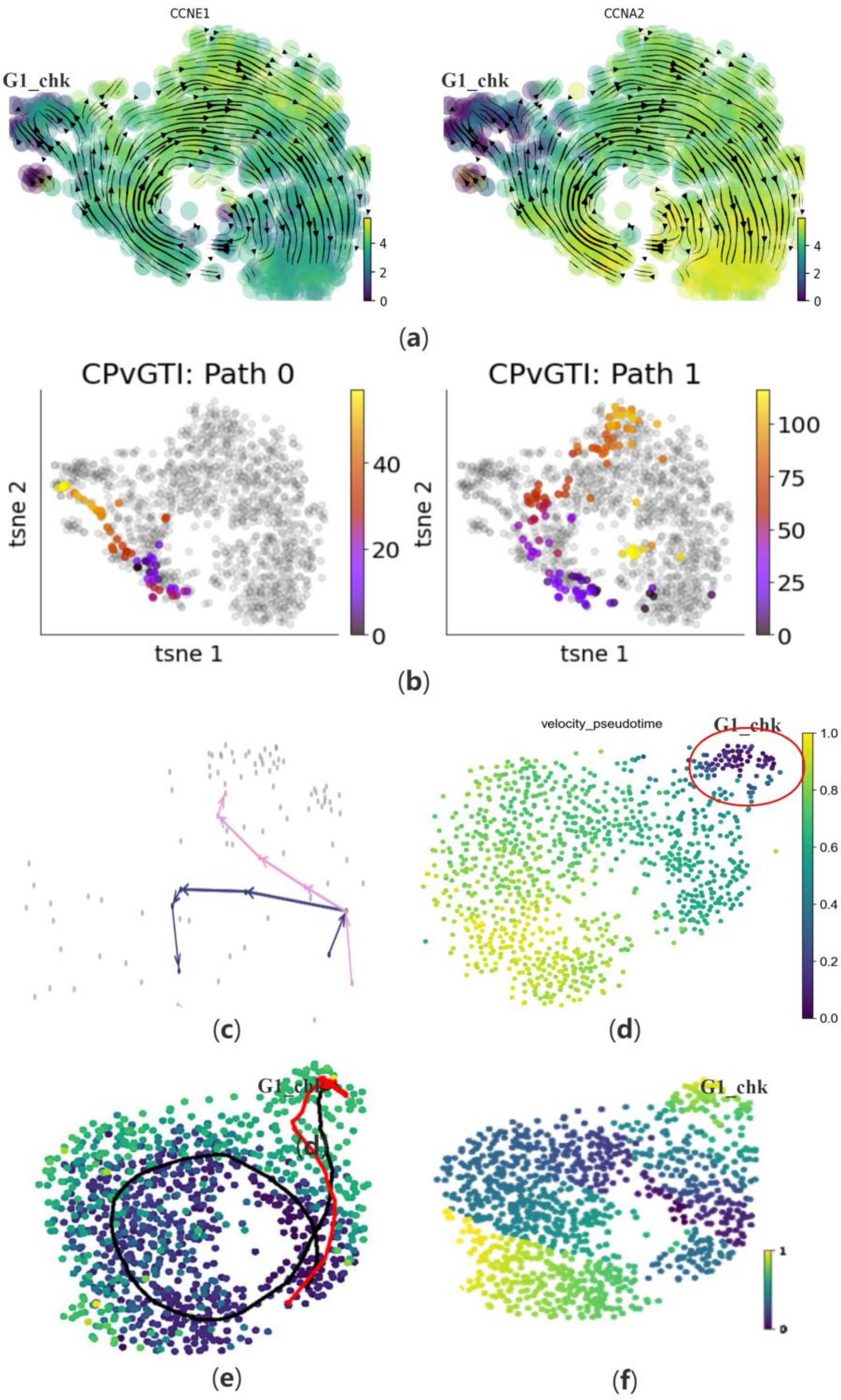

Figure S4. Results of baseline methods on the human cell cycle dataset, related to Figure 6: (**a**) The velocity manifold graph of the marker gene CCNE1 and CCNA2 calculated by scVelo; (**b**) The pseudo-time inferred by CPvGTI; (**c**) The result of CellPath on the human cell cycle dataset; (**d**) Pseudo-time result of LVPT on the human cell cycle dataset; (**e**) The result of CytoPath on the human cell cycle dataset; (**f**) Pseudo-time result of Vetra on the human cell cycle dataset.
